# Microstructural Development and Rheological Study of a Nanocomposite Gel Polymer Electrolyte Based on Functionalized Graphene for Dye-Sensitized Solar Cells

**DOI:** 10.3390/polym12071443

**Published:** 2020-06-27

**Authors:** Pedram Manafi, Hossein Nazockdast, Mohammad Karimi, Mojtaba Sadighi, Luca Magagnin

**Affiliations:** 1 Mahshahr Campus, Amirkabir University of Technology, Mahshahr P.O. Box 63517-13178, Iran; pedram_manafi@aut.ac.ir; 2 Department of Polymer Engineering and Color Technology, Amirkabir University of Technology, Tehran P.O. Box 15875-4413, Iran; 3 School of Materials and Advanced processes Engineering, Department of Textile Engineering, Amirkabir University of Technology (Tehran Polytechnic), Tehran P.O. Box 15875-4413, Iran; mkarimi@aut.ac.ir; 4 Department of Mechanical Engineering, Amirkabir University of Technology, Tehran P.O. Box 15875-4413, Iran; mojtaba@aut.ac.ir; 5 Department of Chemistry, Materials and Chemical Engineering “Giulio Natta,” Politecnico di Milano, 20131 Milano, Italy

**Keywords:** dye-sensitized solar cells, quasi-solid-state electrolyte, functionalized graphene, microstructure of polymer electrolyte, photovoltaic performance

## Abstract

For a liquid electrolyte-based dye-sensitized solar cell (DSSC), long-term device instability is known to negatively affect the ionic conductivity and cell performance. These issues can be resolved by using the so called quasi-solid-state electrolytes. Despite the enhanced ionic conductivity of graphene nanoplatelets (GNPs), their inherent tendency toward aggregation has limited their application in quasi-solid-state electrolytes. In the present study, the GNPs were chemically modified by polyethylene glycol (PEG) through amidation reaction to obtain a dispersible nanostructure in a poly(vinylidene fluoride-co-hexafluoro propylene) copolymer and polyethylene oxide (PVDF–HFP/PEO) polymer-blended gel electrolyte. Maximum ionic conductivity (4.11 × 10^−3^ S cm^−1^) was obtained with the optimal nanocomposite gel polymer electrolyte (GPE) containing 0.75 wt% functionalized graphene nanoplatelets (FGNPs), corresponding to a power conversion efficiency of 5.45%, which was 1.42% and 0.67% higher than those of the nanoparticle-free and optimized-GPE (containing 1 wt% GNP) DSSCs, respectively. Incorporating an optimum dosage of FGNP, a homogenous particle network was fabricated that could effectively mobilize the redox-active species in the amorphous region of the matrix. Surface morphology assessments were further performed through scanning electron microscopy (SEM). The results of rheological measurements revealed the plasticizing effect of the ionic liquid (IL), offering a proper insight into the polymer–particle interactions within the polymeric nanocomposite. Based on differential scanning calorimetry (DSC) investigations, the decrease in the glass transition temperature (and the resultant increase in flexibility) highlighted the influence of IL and polymer–nanoparticle interactions. The obtained results shed light on the effectiveness of the FGNPs for the DSSCs.

## 1. Introduction

Since the introduction of dye-sensitized solar cells (DSSCs) by O’Regan and Grätzel [1] in the early 1990s, the efficiency of the energy conversion has been continuously improved to reach an all-time high of 14% for liquid electrolyte (LE)-based cells [2]. Despite their lower efficiency compared to their counterparts (e.g., 25% for the silicon-based solar cells [3]), DSSCs are still considered as a potential substituent for Si-based photovoltaic cells, thanks to their environmental friendliness and cost-effectiveness. The electrolyte is the most essential part of a DSSC, playing a pivotal role in its function by providing the required medium for charge transport. A majority of high-efficiency DSSCs are based on liquid electrolytes. The extensive use of DSSCs is, however, restricted by several phenomena such as degradation, leakage, dye desorption, thermal instability, and electrode corrosion, mostly due to the application of the conventional LEs [4]. In this regard, numerous studies have been devoted to substituting the conventional LEs with proper alternatives, like ionic liquids [5,6], solid-state perovskites [7], and solid/quasi-solid-state polymer gels and their composites [8,9]. 

Owing to their excellent ionic conductivity, gel polymer electrolytes (GPEs) have been interesting materials in a number of different fields. Generally speaking, a GPE is composed of a polymer base, salt, and a solvent/ionic liquid (IL), where the polymer entraps the IL, the salt provides free ions to enhance the conductivity, and the solvent/IL dissolves the salt and serves as a conducting medium. The polymer not only provides the required mechanical stability, but also serves as a gelation agent [10]. 

The brilliant performance of the PVDF–HFP/PEO as a blend membrane for electrochemical devices has branded it as an applicable material for DSSCs. Owing to the considerable electronegativity and small ionic radius of the fluorine (F), the PVDF–HFP exhibits a slow recombination rate but high ionic conductivity along the interface between the semiconductor photoanode and the polymer electrolyte in a DSSC [11]. As of present, the PEO-based electrolytes have been used to boost the ion exchange through two mechanisms: (a) cation complexation and (2) high-rate diffusion of the ion carriers into the amorphous region of the membrane [12,13]. Therefore, blending of these components will lead to higher ionic conductivity by combining the advantages of the polymers, especially in terms of the higher mechanical stability of PVDF–HFP, with the higher diffusion rate of the ion carriers in the amorphous domain of the PEO. The improvement in ionic conductivity of the blend is mainly caused by the reduced crystallinity and generation of the ion pathways [14].

Electrochemical properties of a gel polymer electrolyte can be improved by adding a filler. Primarily, fillers are selected for the following reasons: (a) reducing the crystallinity and self-aggregation along polymer chains, leading to a more amorphous phase for the gel polymer electrolyte; (b) enhancing salt dissociation while increasing the number of charge carriers; (c) developing a more conductive pathway to improve ionic conductivity; (d) enhancing the stability and mechanical strength of the polymer electrolyte [15,16]. Therefore, the size [17] and content [18] of the filler nanoparticles (NPs) as well as their interaction (chemical/physical or interface/interphase) with the various constituents could dramatically affect the final properties of the nanocomposite. Graphene nanoplatelets (GNPs) have exhibited large potentials, thanks to their excellent mechanical, thermal and electrical features as well as their capability of forming interconnected networks at relatively low contents. Such improvements in characteristics at such low contents have been assigned to the high aspect ratio of the GNPs [19]. Yet, fine dispersion and distribution of carbonaceous NPs in the polymer matrix have remained unsolved issues [20,21]. Homogeneous dispersion of carbonaceous NPs in the polymer can be enhanced by chemical or physical functionalization [22,23]. Many studies have been published about polymeric electrolytes containing GNP and FGNP [24,25,26,27]. Marchezi et al. [28] prepared a gel polymer electrolyte composed of PEO, γ-butyrolactone (GBL), LiI, I_2_, and different concentrations of reduced graphene oxide (RGO). Upon using only 0.5 wt% of RGO, they achieved an efficiency of 5.07% with the highest short-circuit photocurrent (J_SC_) and open-circuit potential (V_oc_). They suggested that NPs acted as a multifunctional component in the GPE. In a very recent study, Gomari et al. [29] pioneeringly grafted poly ethylene glycol (PEG) onto graphene and employed it in a PEO electrolyte to enhance ionic conductivity through lowering the nanocomposite crystallinity. According to their results, PEG-grafted graphene was achieved through hydrogen bonding with oxygen atoms of PEO chains, possibly deteriorating the crystallinity of the PEO-based electrolyte. PEG-grafted graphene could contribute in either of two ways: enhancing the segmental motion of PEO chains for higher flexibility and promoting better distribution through the polymer matrix. Recently, Prabakaran et al. [30] prepared a polymer electrolyte based on PVDF–HFP/PEO (60/40 wt/wt) and 0.8 wt% RGO, ending up with a maximal efficiency of 4.6%. Rehman et al. [31] reported a DSSC using a polyvinyl acetate (PVAc)/graphene nanocomposite-based gel electrolyte, which could realize a high efficiency of 4.57%.

The microstructure of GPE plays a vital role in the final performance of the device. Concerning the blend, the miscibility of two components in the amorphous domain can result in homogenous pathways for ion carriers [32]. In the case of nanocomposites, the viscoelasticity and rheological features are highly dependent on the distribution of nanofillers in the polymer matrix. These properties could also help better understand the molecular interactions occurring between the filler and the polymer matrix, and hence, provide guidelines for improving the performance of electrochemical devices [33,34]. 

Composed of organic cations and counter-ions, ILs have drawn a considerable deal of attention as alternatives to organic solvents, making them capable of playing an important role in a GPE system. Muhammad et al. [35] prepared a DSSC with 8 wt% IL (1-methyl-3-propylimidazolium iodide, i.e., MPImI) in polymer electrolytes based on hexanoyl chitosan/poly(vinyl chloride) (PVC), and the device performed at an efficiency (*η*) of 4.55%. The noncovalent interactions of CNTs with a polymer matrix (e.g., polyurethane and PVDF) in the presence of an IL leads to the fine dispersion and distribution of the NPs, bringing about some synergistically improved properties. Among the crystalline and amorphous phases of GPEs, the amorphous phase is significantly related to ionic conductivity [36]. According to Vyas and Chandra, EMIMBF_4_ is an ionic liquid capable of providing free ions and increasing the amorphous phase of the polymer in the presence of single-walled carbon nanotubes (SWCNT), hence, serving as a plasticizer and giving rise to enhanced ionic conductivity [37]. 

Given the mentioned concerns regarding the DSSCs, this study is aimed at increasing the efficiency of the device through developing a novel GPE. An attempt was also made to investigate the effect of PEG-grafted graphene on the microstructure and hence the ionic conductivity and conduction mechanism of the GPE. A miscible PVDF–HFP/PEO mixture was used as the basic GPE blend, while an ionic liquid served as the plasticizing agent to enhance the ionic conductivity of the GPE. Accordingly, the graphene was functionalized by PEG in the presence of a carbodiimide condensing agent to prepare the gel polymer electrolyte. Then, functionalized graphene was added to the PVDF–HFP/PEO blend at various contents to investigate the effect of the functionalized graphene dosage on ionic conductivity and DSSC performance. The microstructure and its relationship with ionic conductivity and electrochemistry properties of nanocomposite GPEs were thoroughly investigated by a combination of thermal and rheological studies and SEM and impedance analyses. To the best of our knowledge, this blend and functionalized graphene have not been addressed as an electrolyte.

## 2. Experimental Section

### 2.1. Materials

The semi-crystalline polar block copolymer of PVDF–HFP (average *M_w_*~4 × 10^5^ g mol^−1^, average *M_n_*~1.3 × 10^5^ g mol^−1^ with PDI 3.077) and PEO powder (average M_v_ of 6 × 10^5^ g mol^−1^) were employed as the host polymer matrix. PEG (M_W_ = 600 g mol^−1^), N,Ndicyclohexylcarbodiimide (DCC) as a dehydrating agent, anhydrous dimethyl formamide (DMF) (99.8%), and 1-butyl-3-methylimidazolium tetrafluoroborate (BMIMBF_4_) as IL were applied. Lithium tetrafluoroborate (LiBF_4_) (molar mass: 93.746 g mol^−1^) was employed, owing to its low lattice energy and high dissolution power. Anhydrous Lithium Iodide (LiI) and Iodine (I_2_) were applied as a redox couple, which is responsible for dye regeneration. All chemicals used in this study were supplied from Sigma-Aldrich (Taufkirchen, Germany). Fluorine-doped tin oxide (FTO) glass (with surface resistivity of 7 Ω cm^−2^) and cis-di-isothiocyanato-bis(2,2’-bipyridyl-4,4’-dicarboxylato)-ruthenium(II)-bis(tetrabutylammonium) (N-719) dye were procured by Solaronix, Aubonne, Switzerland. Angstron Materials (Dayton, OH, USA) supplied graphene nanoplatelets (GNP, N002-PDR) with thickness and average diameter values below 1 nm and 10 μm, respectively. Average aspect ratio and surface area of the GNPs were respectively 4000 and 400–800 m^2^ g^−1^, with stacks of 1–3 monolayer graphene sheets. 

### 2.2. Preparation of PEG-Grafted Graphene

**Figure 1:**
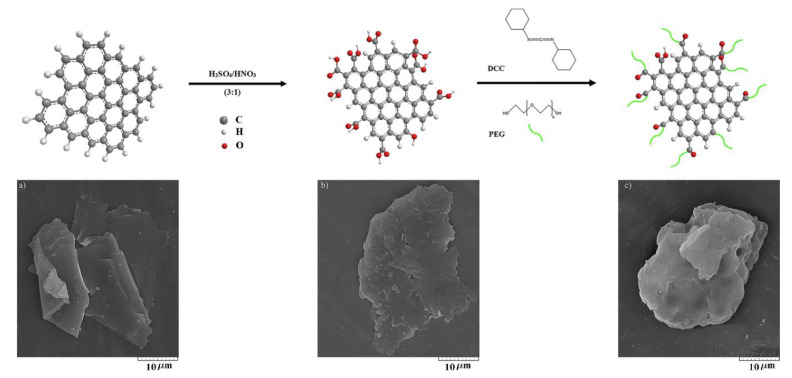
SEM micrographs of (**a**) GNP, (**b**) GNP–COOH, and (**c**) FGNP. The inset shows the steps of the functionalization reaction for the graphene nanoplatelets.

To remove the adsorbed humidity, 0.2 g of GNP was vacuum-dried at 80 °C for 72 h. The GNPs were oxidized by a mixture of H_2_SO_4_/HNO_3_ (3:1, v/v) at 55 °C for 2 h under ultrasonic treatment to carboxylate the graphene (GNP–COOH). The carboxylated GNP was then filtered by polycarbonate with a pore diameter of 0.45 μm followed by washing several times with deionized water, until its pH reached 7. It was then subjected vacuum-drying at 80 °C for 24 h. Afterward, GNP–COOH was reacted with PEG600 through carbodiimide-activated esterification reaction [38]. Subsequently, 0.2 g of GNP–COOH, 0.8 g of PEG600, and 0.02 g of dried DCC were added to 50 ml of anhydrous tetrahydrofuran (THF) at 60 °C under stirring and refluxing by nitrogen atmosphere for 48 h. The unreacted PEG was removed by complete washing with dried THF, absolute ethanol, and deionized water followed by filtration through poly(tetrafluoroethylene) (with a pore diameter of 0.45 μm). The final product was obtained after vacuum drying at 60 °C for 24 h and tightly sealed in a bottle. The aforementioned steps are shown in the inset of Figure 1.

### 2.3. Preparation of Gel Polymer Electrolyte

In order to synthesize an ion-conducting polymer film, appropriate amounts of PVDF–HFP and PEO (at the optimal PVDF–HFP:PEO ratio of 60:40 wt%) were dissolved in DMF (at a concentration of 15 wt%) at 80 °C for 4 h using a magnetic stirrer. Afterward, as a plasticizing agent, BMIMBF_4_ was added and the mixture was further stirred for 6 h. The content consistency was maintained in all formulations. The ionic conductivity was further enhanced by incorporating LiBF_4_ salt and LiI/I_2_ (a as redox couple) under continuous stirring for 6–8 h until a viscous solution was obtained. The GPE-containing liquid medium (i.e., containing 1 g of 70: 10/1: 20/1, PVDF–HFP/PEO: BMIMBF_4_/LiBF_4_: LiI/I_2_) offered the maximal ionic conductivity (σ = 2.15 × 10^−3^ S cm^−1^) at room temperature. Ultimately, the viscous solutions were cast into a petri dish to dry at 80 °C. After peeling off the dried gels, a free-standing and flexible thin film was attained, which was ready for future tests. 

The performance was further enhanced by synthesizing the ion–electron-conducting polymer composites with various contents of GNP and FGNP in PVDF–HFP/PEO: BMIMBF_4_/LiBF_4_:LiI/I_2_ polymeric film. Different levels of functionalized or pristine graphene nanoplatelets (i.e., 0.1, 0.25, 0.5, 0.75, 1, or 1.5 wt% graphene relative to blend) were incorporated into the mixture containing BMIMBF_4_: LiBF_4_ at 10:1 *w*/*w* (as an IL complex) and LiI/I_2_ at 20:1 *w*/*w* (as a redox couple) in all samples. The black suspension was ultrasonicated under stirring for 20 min to form a homogenous dispersion of the graphene; it was then incorporated into the PVDF–HFP and PEO mixture (60:40 *w*/*w*). The obtained mixture was further stirred at 80 °C, until complete dissolution of the polymer content, followed by cooling down to the ambient temperature, which trigged the gelation process. At room temperature, the largest ionic conductivities (σ = 3.95 × 10^−3^ S cm^−1^ and σ = 4.11 × 10^−3^ S cm^−1^) were observed in nanocomposite membranes containing GNP and FGNP (1 and 0.75 wt%, respectively); thus, they were selected as the optimal samples. The obtained GPEs were directly applied in electrochemical experiments with no further treatments. The sample codes and their composition are listed in Table 1.

### 2.4. Protocol of Dye-Sensitized Solar Cells Assembly

The colloidal suspensions were obtained by grinding 0.25 g of TiO_2_ (Evonic P90 Aeroxide) and 0.1 M HNO_3._ A layer including fine TiO_2_ particles (∼14 nm) was coated on the pre-cleaned FTO glass plate by the doctor blade method. The samples were then sintered at 450 °C for 45 min followed by slow cooling down to room temperature. The FTO glass plates were cut in ∼1 cm × 1 cm pieces and half of them were coated with a scotch tape to avoid TiO_2_ deposition on the electrical contact areas. Thus, the TiO_2_ layer covered ∼ 0.25 cm^2^ of the cell. The other TiO_2_ colloidal suspension was obtained through mixing 0.25 g of TiO_2_ (Degussa P-25), 0.1 M nitric acid, 0.02 g of triton X-100, and 0.05 g of PEG1000. The prepared paste was used to coat the former TiO_2_ layer using the doctor blade method to achieve a photoanode with a large particle size and an active cell area of 0.25 cm^2^. The two-layer TiO_2_ photoanode was resintered at 450 °C, followed by cooling down to ambient temperature. The dye-sensitized TiO_2_ photoanode electrode was fabricated by immersing the samples in 0.5 mM N-719 ethanolic dye solution for 24 h to adsorb the dye. To produce the counter electrode (cathode), one drop of 5 mM chloroplatinic acid hexahydrate (H_2_PtCl_6_) was dissolved in isopropyl alcohol and spread over the conductive surface of FTO, followed by a 15 minute calcination stage at 450 °C under an atmosphere of air. A viscous solution of each GPE sample was spilled onto the photoanode and kept there for 24 h to form a proper GPE before cleaning the excess gel electrolyte from the non-active surface area of the photoanode. Finally, DSSCs were fabricated with the following configuration: FTO/TiO2/N719/GPE/Pt/FTO.

### 2.5. Characterization

The functionalization process was characterized by FTIR analysis using a Bruker EQUINOX 55 FTIR spectrometer (Ettlingen, Germany), collecting 32 scans in the 400–4000 cm^−1^ range at a resolution of 4 cm^−1^. In a typical procedure, graphene samples were ground with dry KBr and compressed into pellets. In the case of the PEG, a thin layer of the diluted solution was prepared on KBr pellets. Raman analysis was conducted by ALMEGA-Dispersive Raman, Thermo Nicolet (Madison, WI, USA) with an excitation wavelength of 514.5 nm. TGA was also carried out using a Polymer Laboratories model STA 1640 (Shropshire, UK) under N_2_ atmosphere from room temperature to 600 °C at 10 °C min^−1^ for monitoring GNP modification. Scanning electron microscopy (SEM) was done on a Tescan VEGA-II SEM (Brno, Czech Republic) equipped with an energy-dispersive X-ray (EDX) analysis module operating at 20 kV. Elemental analysis of the samples was performed by an Elementar Vario EL III (Langenselbold, Germany) to confirm the functionalization reaction. The pore size of the membrane films was measured via two approaches, once by utilizing the mercury intrusion porosimetry (MIP) (Micromeritics Poresizer model 9320) technique, and another time by the Brunauer–Emmett–Teller (BET) analysis using a Gemini VII 2390a (Micromeritics, Norcross, GA, USA) with N_2_ as the sorbate. Differential scanning calorimetry (DSC) was done in the temperature range of −70 to 200 °C using a TA Instrument DSC 2010 thermal analyzer (New Castle, Delaware, USA). The oscillatory shear rheological assessment was performed at 190 °C using an MCR 301 Rheometer (Physica Anton Paar, Graz, Austria) with a parallel-plate geometry (with a diameter and gap of 25 and 1 mm, respectively). The dynamic strain amplitude sweep experiment was conducted to determine the linear viscoelastic region through storage modulus analysis.

Ionic conductivities of the GPEs were determined by electrochemical impedance spectroscopy (EIS) (Bio-Logic SAS, VSP-300, Grenoble, France) at 25 °C and an AC voltage of 10 mV in the frequency range of 1 mHz to 1 MHz, where the GPE specimens were sandwiched by two stainless steel blocking electrodes (area, A = 0.785 cm^2^). The following equation was used to measure the ionic conductivity of the investigated electrolyte, with the results based on the Nyquist plot [39]:

(1) $$\sigma =\frac{d}{R_{b}A}$$

where d and R_b_ are thickness and bulk resistance of the membrane discs, respectively, and A indicates the area of the electrode in contact with the electrolyte membrane.

Temperature-dependent EIS assessments were addressed at 30–100 °C at a step size of 10 °C. The effective diffusion coefficient of ionic species ($D_{app}^{I_{3}^{-}}$) in GPEs was obtained through steady-state linear sweep voltammetry (LSV) tests with a potentiostat (Bio-Logic SAS, VSP-300, France) at a scanning rate of 10 mV s^−1^ at ±700 mV and a temperature of 25 °C. The dummy cell applied in the LSV investigations included GPE samples sandwiched by two Pt counter electrodes (area, A = 0.785 cm^2^), whose spacer thickness, l, was 50 μm. 

The photocurrent density was plotted vs. voltage (*J–V*) for the DSSC across the active area of 0.25 cm^2^ using an ABET SunLite solar simulator 11002 (Milford, Connecticut) equipped with a 100 W Xenon arc lamp within the irradiation voltage range of +1 to −1 V. A mask was used to block the light outside the active area to avoid the collection of extra current. The *J–V* plots were determined in the linear sweep mode at a scan rate of 100 mV s^−1^. The *J*–*V* curve was used to calculate the photovoltaic indices like *J_sc_*, *V_oc_*, *FF*, and *η*%. The fill factor (*FF*) and efficiency (*ɳ*) were calculated by:

(2) $$FF=\;\frac{P_{max}}{V_{oc}\cdot J_{sc}}$$

(3) $$ɳ\left(\%\right)=\frac{V_{oc}\cdot J_{sc}\cdot FF}{P_{in}}\times 100\%$$

wherein *P_max_* (mW cm^−2^) is the maximum power output of the DSSC, *V_oc_* denotes the open-circuit voltage, *J_SC_* stands for the short circuit current density and *P_in_* represents the incident light power. 

EIS data of DSSCs were determined in the frequency range of 0.1 Hz to 100 kHz at an open-circuit potential and perturbation amplitude of 10 mV (scan rate: 100 mV s^−1^; bias: 0.55 V) through applying a frequency response analyzer. The Bio-Logic software was employed to fit data to a three-time constant equivalent-circuit model.

## 3. Results and Discussion

### 3.1. Functionalization Reactions

The SEM analysis was employed to monitor the morphology of graphene nanoplatelets during the functionalization process, as shown in Figure 1. Herein investigated is the effects of utilizing PEG-grafted GNPs as filler for augmenting the performance of the GPEs in DSSCs. The main aim of using the SEM analysis in this work was to qualitatively evaluate the morphological changes occurred due to oxidation and functionalization reactions. The grafting of PEG is designed to mitigate the aggregation tendencies of GNPs that are known to lead to poor dispersibility in the matrix. Inappropriate functionalization of the FGNPs would lead to their break-up and rupture, thereby shortening their effective length, and hence, lowering the ionic conductivity [40]. As seen in Figure 1a, the diameter of the smooth surface of pristine GNP is around 10 μm. After oxidation of the GNP (Figure 1b) and then grafting of the PEG on the GNP–COOH (Figure 1c), neither of them showed highly aggregated structures. Furthermore, the graphene exhibited no noticeable damage or alteration in the average diameter following the functionalization reaction.

**Figure 2:**
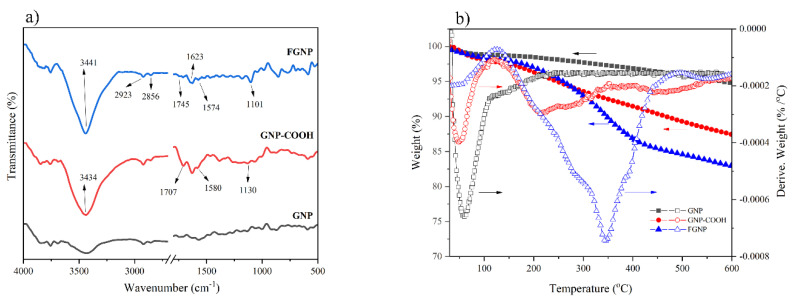
(**a**) FTIR spectra, and (**b**) TGA-DTG diagrams of GNP, GNP–COOH, and FGNP.

FTIR spectroscopy has served as an influential tool for studying the functionalization reaction of GNP. Figure 2a presents the FTIR result of the pristine GNP, GNP–COOH, and FGNP. FTIR spectra of the neat PEG600 is shown in Figure S1. For the GNP–COOH sample, the new absorption peaks at 1707, 1580 and 1130 cm^−1^ reflect the creation of a carbonyl group from carboxylic acid (–COOH–) and asymmetric and symmetric stretching vibrations of the –COO–, respectively. The strongest absorption band was observed at 3434 cm^−1^; it could be attributed to the stretching vibration of the –OH in the hydroxyl group. This band was significantly intensified after the treatment. It should be noted that the peaks at 1430–1650 cm^−1^ are related to the stretching vibrations of the C=C on the aromatic rings. The shoulder emerged at ~1580 cm^−1^ originated from untreated aromatic regions of the carboxylic acids in GNP [41]. These are apparent evidence of the acid treatment on the surface of the pristine graphene.

After functionalization with PEG, a new peak appeared at 1101 cm^−1^. This is the characteristic peak of PEG associated with C–O–C ether groups. The peak at 1745 cm^−1^ implied a blue shift for the carbonyl band, suggesting the formation of ester links [42]. The peaks at 2856 and 2923 cm^−1^ represent the C–H stretching of alkyl groups in the PEG. The peak at 3441 cm^−1^ got enhanced by the hydroxyl absorption band in relation to the end groups of the PEG. Remarkably, the intensification of the peak at 1623 cm^−1^ and the weakening of the one at 1574 cm^−1^ reflect the chemical reaction of PEG chains with COOH groups of the oxidized graphene. Thus, it can be concluded that FTIR spectra confirm the chemical grafting of the PEG onto the GNPs (see the Supporting Material for details on the functionalization reaction of GNP by Raman analysis (Figure S2)).

Thermal gravimetric analysis (TGA) was performed to quantitatively investigate the GNP functionalization reaction. Figure 2b illustrates the variations of weight percentage with temperature for the pristine GNP, GNP–COOH and FGNP samples. At 600 °C, the weight of the residue was 94.7, 87.45, and 82.95 wt% for pristine GNP, GNP–COOH, and FGNP, respectively. Obviously, the functionalized samples were more sensitive to the weight loss, so the pyrolysis of the functional groups occurred at lower temperatures compared to the pristine GNP. Such difference could be attributed to the attachment of the functional groups to the NPs. The one-step degradation that occurred at ~60–100 °C corresponded to the evaporation of the physio-adsorbed water and the release of moisture. At 160–345 °C, the observed weight loss could be assigned to the pyrolyzed liable oxygen-containing functional groups [43]. As can be seen on the derivative spectra of GNP–COOH and FGNP, appearing at some temperatures below 100°C, the first peak corresponded to the release of moisture; the second one could be attributed to the pyrolysis of the liable oxygen-bearing functional groups, resulting in some 5% weight loss at 245 °C. As expected, the second-stage weight loss (~11% at 355 °C) of the FGNP occurred at higher temperatures because of the thermal resistance of the PEG chains [44]. Similar results were reported by our group [21] (see the Supporting Material for details on quantitative determination of the GNP functionalization reaction by EDX and CHNO elemental analysis (Table S1)).

### 3.2. Differential Scanning Calorimetry

**Figure 3:**
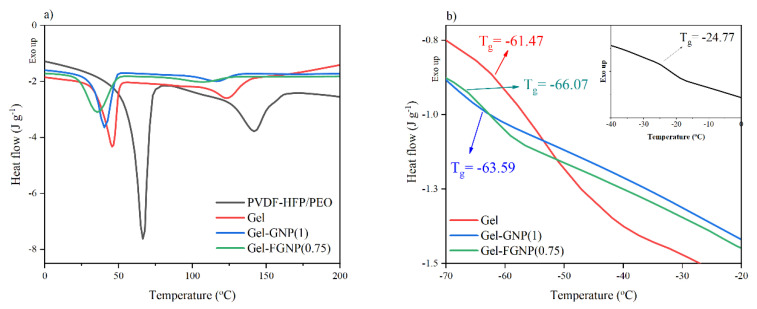
(**a**) DSC thermograms of PVDF–HFP/PEO, Gel, Gel–GNP(1), and Gel–FGNP(0.75) samples, (**b**) shows glass transition temperature (T_g_)).

Figure 3 presents the DSC results of the PVDF–HFP/PEO, Gel, Gel–GNP(1), and Gel–FGNP(0.75) composite films in the temperature range of −70 to 200 °C. The endothermic peaks at 140.40 and 66.58 °C indicate the melting points (*T_m_*) of the PVDF–HFP and PEO blend components, respectively. Due to the differences in crystallization characteristics, including *T_c_*, *T_m_*, and degree of crystallinity of the components, the PVDF–HFP/PEO blend underwent a crystallization-induced phase separation (Figure 3a). In other words, their blend encompassed two separate crystallization domains along with a single miscible amorphous phase. This was also the case for other samples containing IL and NPs. Incorporation of the IL into the blend decreased the melting points of the PVDF–HFP and PEO to 123.11 and 45.64 °C, respectively. It should be noted that the decrease in the *T_m_* of the Gel sample could be related to the partial diffusion of the IL into the crystalline phase as a result of the strong interactions between the IL and polymer blend molecules. The melting points of the PVDF–HFP and PEO showed obvious shifts toward 115.68 and 40.41 °C in the presence of the GNPs, respectively. Their melting points were also shifted to 110.48 and 36.73 °C in the presence of the FGNPs in the Gel–FGNP(0.75), in the same order. The larger drop of *T_m_* of the blend components with the FGNPs could be assigned to the increased defect density within the crystallites of the blend components because of the improved dispersion and stronger chain interaction with the FGNPs. It should be noted that further addition of either GNP or FGNP may result in their aggregation, thereby increasing the crystallinity of the samples (Table S2).

Moreover, the degree of crystallinity and enthalpy of the GPEs play a decisive role in the ionic conductivity of the electrolyte. In this regard, the degree of crystallinity (χ%) and enthalpy of the GPEs were determined, as listed in Table 2.

Clearly, IL contributed as a plasticizer and increased the free volume of the matrix, leading to a drastic reduction in the enthalpy and crystallinity, hence, improving the ionic conductivity. Indeed, the crystallinity and enthalpy of the samples were reduced by the addition of functionalized graphene (up to 0.75%). The high surface area of FGNPs dramatically disrupt the dense molecular package of the polymer chains and enhanced the amorphous phase. Furthermore, thanks to its intensive interfacial interactions with the blend components, the PEG-grafted graphene helps achieve a proper dispersion of the NPs. Thus, the increased free space volume in the polymeric nanocomposite can improve the ionic conductivity of the system [45]. Further incorporation of FGNPs, however, triggers the recrystallization of the polymer segments, thereby setting the scene for higher degrees of crystallinity (Table S2).

Glass transition temperature (*T_g_*) of a polymer is an indication of polymer chain mobility. This index was also assessed, as presented in Figure 3b. In comparison with GNP, the FGNP had a higher impact on the *T_g_* of the samples, so that the Gel–FGNP(0.75) sample exhibited a *T_g_* of −66.07 °C (3 °C lower than that of the Gel–GNP(1)). This implies that the mobility of PVDF–HFP and PEO chains was improved by incorporation of the FGNP. The disturbance of the crystalline structure and packed molecular configuration as well as enhanced free volume in the matrix have been proposed to explain the decrease in *T_g_* upon adding the NPs [45,46]. In this research, however, the plasticizing impact of the GNP-grafted PEG was also reported as another key reason. Interestingly, the single *T_g_* reflected the good miscibility of the polymer components, IL, and NPs at the applied ratios in all cases within the amorphous domains. However, two distinct peaks emerged at higher temperatures, manifesting a phenomenon called “crystallization induced phase separation”. Therefore, the reduced crystallinity together with the fine miscibility of the GPE components are the two key factors explaining the improved ionic conductivity of the GPEs, which would improve the DSSC performance in turn.

### 3.3. Viscoelastic Characteristics of Gel Polymer Electrolytes

**Figure 4:**
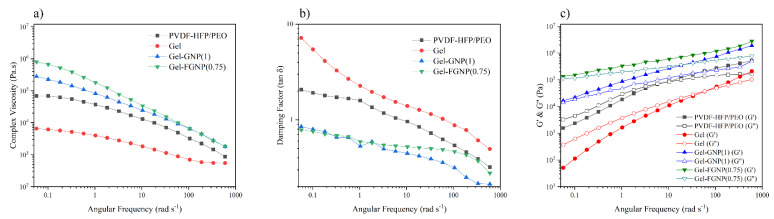
(**a**) complex viscosity, (**b**) damping factor against the angular frequency, and (**c**) storage and loss moduli of PVDF–HFP/PEO, Gel, Gel–GNP(1), and Gel–FGNP(0.75) at 190 °C.

In order to further understand the microstructural properties of the polymeric matrix and nanofillers and elucidate the influence of the NPs’ dispersion, the linear viscoelastic behavior of the membrane electrolytes was investigated using the so called melt rheology, as shown in Figure 4 (see Figure S3 for differentiating the linear viscoelastic region from the nonlinear).

The major viscoelastic features in low-amplitude oscillations were evaluated in terms of frequency. Figure 4a–c illustrates the changes in the complex viscosities (η*), loss factor (tan *δ*), and storage and loss moduli in the range of 0.05–600 rad s^−1^. According to Figure 4a, the frequency-dependent viscoelastic studies showed a reduction in the viscosity of the gel polymer electrolyte (Gel) upon the incorporation of IL into the polymeric matrix, which might be ascribed to its liquid nature or plasticizing influence. The fact that NPs’ incorporation enhanced the viscosity indicates the interconnected or network-like structure of (F)GNPs. The introduction of FGNPs increased the viscosity to higher levels than that achieved with the GNPs. The higher viscosity of Gel–FGNP(0.75) might be due to the better dispersion of NPs within the matrix. In other words, the nanocomposite samples offered enhanced viscosity upturn and reinforced nonterminal (also known as solid-like) characteristics at lower frequencies, which are indicative of the creation of a particle–particle and/or particle–matrix network. The strength of the mentioned networks or their extent of formation increased by improving the dispersion through adding the FGNPs and increasing the nanofiller concentration. Thus, an interfacial interaction can be concluded between the PVDF–HFP/PEO matrix and the nanoplatelets. It should be noted that the decrease in η* with increasing the frequency is a manifestation of the pseudoplastic behavior of the polymer nanocomposites due to the random orientation of the entangled nanoplatelets [47]. 

Substance viscoelasticity can be evaluated by its damping/loss factor (tan *δ* = *G*′′/*G*′), where smaller damping factors are indicative of the solid-like behavior. Figure 4b presents the variations of tan *δ* with angular frequency for PVDF–HFP/PEO, Gel, Gel–GNP(1), and Gel–FGNP(0.75). Compared to the pure blend, the Gel sample showed a more viscous behavior due to its greater tan *δ* (tan *δ* > 1). Inclusion of nanoplatelets into the Gel sample decreased the damping factor, suggesting a solid-like viscoelastic nature for the sample (tan *δ* < 1). This phenomenon can be attributed to the interfacial bonding between the nanoplatelets and the polymer chains. Thus, the chains’ movements will be restricted, resulting in a prolonged relaxation time. Such an observation was more profound in the FGNP-based samples [48].

Figure 4c shows the variations of storage and loss moduli with the angular frequency for PVDF–HFP/PEO, Gel, Gel–GNP(1), and Gel–FGNP(0.75). As the frequency enhanced, the moduli increased, since the polymer chains lacked enough time to relax at higher frequencies. Besides, lower frequencies or longer times promoted polymer chain relaxation and hence, lowered the moduli. Moreover, nanoparticle incorporation increased the blend moduli by promoting the graphene nanoplatelets–polymer chain interactions. Indeed, the NPs improved the viscoelastic features of the polymers by their superior stiffness [49]. Intersections of the G′ and G″ were observed at 7.82 and 58.74 rad s^−1^ for the PVDF–HFP/PEO and Gel samples, respectively, implying a shorter deformation relaxation time for the Gel sample rather than the blend. For the Gel sample, the crossover point was detected at ~58.74 rad s^−1^, below which *G*′′ > *G*′ and above which *G*′ > *G*′′. For the former range, the imposed mechanical force exceeds the attractive molecular forces; thus, the material initiates to yield. Above 58.74 rad s^−1^, this is the attractive molecular force (between the IL and PVDF–HFP/PEO) that exceeds the imposed mechanical force (at 190 °C). The liquidus nature of BMIMBF_4_ alters the crossover frequency to a higher frequency than that with the PVDF–HFP/PEO. The IL-containing polymer exhibited liquid-like features, i.e., *G′* < *G*″, at low angular frequencies (<58.74 rad s^−1^), while at frequencies higher than 58.74 rad s^−1^, it offered solid-like characteristics, i.e., *G′* > *G*″. However, the nanocomposite samples demonstrate no G′–G″ intersection at lower frequencies, as the robust nanoparticle networks altered the chain deformation relaxation toward very small frequencies (very long times). This tendency got more intensified in the FGNP-containing samples. To say it differently, the deformation relaxation of the chains is not measurable in the tested range of frequencies, as it occurs in a very long time. Furthermore, according to Figure 4c, *G*′ is higher than *G*′′ in the nanocomposite samples all along the considered frequency range. 

From another perspective, at lower frequencies, the neat blend and Gel samples exhibited the classic terminal viscoelastic features, with the scaling of *G*′ ∼ *ω*^2^. The storage modulus of the pristine GNP-containing nanocomposites slightly deviated from the classic regime in the range of lower frequencies. Nonetheless, the oscillatory characteristics of the functionalized FGNP-based nanocomposites were different. For the mentioned samples, the storage modulus exhibited a nonterminal plateau (i.e., solid-like feature) at lower frequencies and offered greater values in comparison with the neat PVDF–HFP/PEO, Gel, and Gel–GNP(1). These findings indicate a relatively proper nanoparticle dispersion because of the reinforced interfacial interactions between the matrix and the FGNP in comparison with the GNP. At higher frequencies, the storage moduli of all samples were similar to that of the neat PVDF–HFP/PEO, implying the decisive role of the segmental motion of the PVDF–HFP/PEO matrix in its response for shorter times. For more details, the rheology parameters of the neat PVDF–HFP, neat PEO, and PVDF–HFP/PEO blend at 190 °C are shown in Figure S4. In this Figure, Palierne’s model is not fitted, which means that the system has its interfacial tension unchanged, with the miscible state in the molten phase remaining in the blend [50].

### 3.4. Morphology of the Gel Polymer Electrolytes

**Figure 5:**
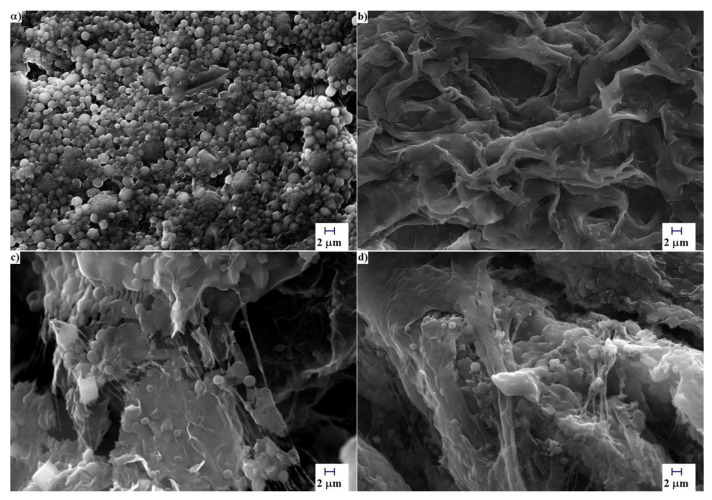
SEM micrographs of (**a**) PVDF–HFP/PEO, (**b**) Gel, (**c**) Gel–GNP(1), and (**d**) Gel–FGNP(0.75).

Figure 5 demonstrates the morphological properties of the PVDF–HFP/PEO, Gel, Gel–GNP(1), and Gel–FGNP(0.75) membrane films. According to Figure 5a, the PVDF–HFP/PEO blend film possessed a rough, granular, uneven surface with evenly distributed crystalline grains across the entire membrane film. Upon introduction of the IL into the polymer matrix, the smoothness of the polymer film was improved, resulting in a flexible film (Figure 5b). In the Gel–GNP(1) and Gel–FGNP(0.75) membranes (shown in Figure 5c,d, respectively), the fact that no nanoplatelets were observed suggested that the polymer matrix had fully wrapped the nanoplatelets, altering the surface morphology. Moreover, the graphene nanoplatelets established a highly porous and interconnected network, with a pore size of 10–15 μm (see pore size determination on Figure S5 and Table S2). It seems that the presence of FGNPs did not cause any significant difference in the pore size, as compared with the GNPs (Figure S6 schematically represents the structural diagrams of four different polymeric membranes).

### 3.5. Electrochemical Performance of Gel Polymer Electrolytes

#### 3.5.1. Ionic Conductivity of Gel Polymer Electrolytes

**Figure 6:**
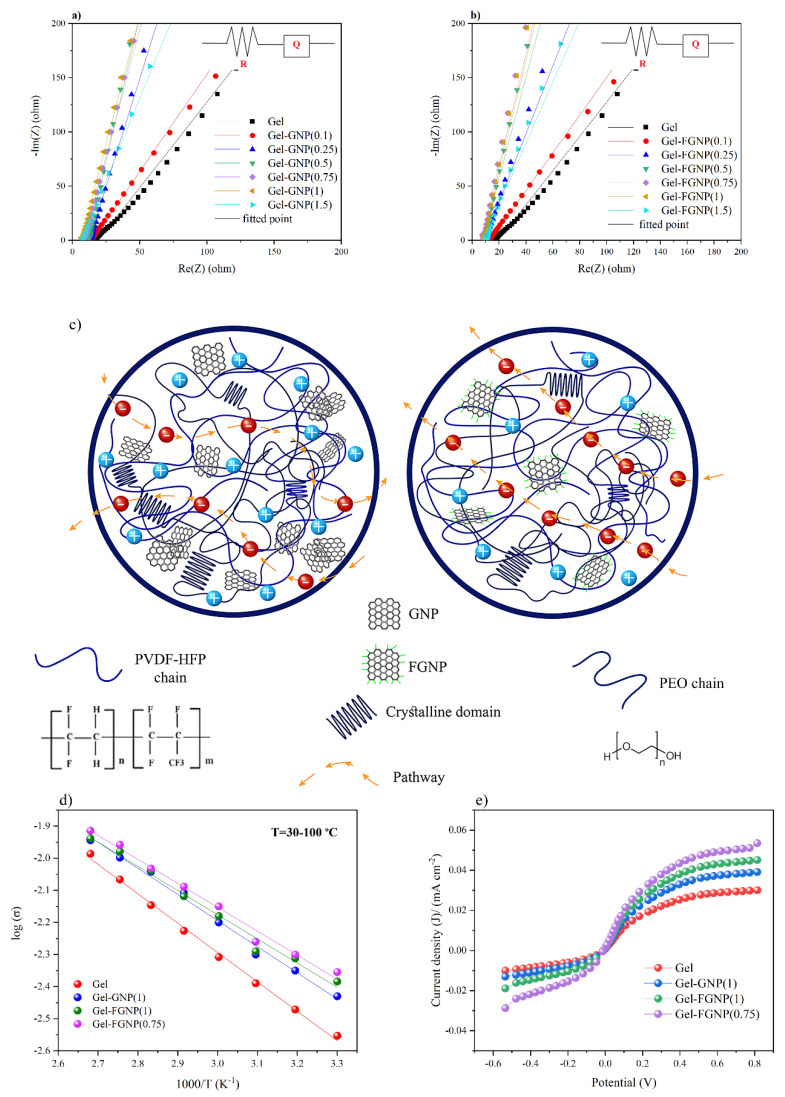
Nyquist plots of (**a**) Gel and Gel–GNP electrolyte membranes containing various amounts of GNPs and (**b**) Gel and Gel–FGNP electrolyte membranes with different amounts of FGNPs. (**c**) Schematic structures of Gel, Gel–GNP, and Gel–FGNP electrolyte membranes. (**d**) Arrhenius plots of GPEs indicating a charge-hopping ion transport mechanism. (**e**) LSV voltammogram and activation energy for Gel, Gel–GNP(1), Gel–FGNP(1) and Gel–FGNP(0.75) electrolytes.

The ionic conductivity of gel electrolyte and graphene-based gel electrolytes was assessed by EIS analysis. Figure 6a–b depicts the typical Nyquist plots developed upon impedance analysis of the Gel, Gel–GNP and Gel–FGNP electrolytes containing graphene at various contents. At higher frequencies, the non-zero intersect on the Nyquist plot of the gel films is indicative of ohmic resistance, while the straight line at lower frequencies reflects a capacitive characteristic. Van Schalkwijk and Scrosati suggested that any deviation from linearity across the whole frequency range of a Nyquist plot (e.g., the arc form at lower frequencies) might indicate either the immiscibility of the polymer blend and/or the presence of highly crystalline domains that were absent in the nanocomposite samples. It should be noted that ions can only migrate through the amorphous phase. The Gel sample exhibited a slight deviation from linearity [51]. The impedance spectrum of the gel membranes was assessed using the Bio-Logic software based on the electrical equivalent circuit (the intersection on Figure 6a,b). Herein, the parameter *R* denotes the electrolyte ohmic resistance and Q represents the constant phase element of the double layer. *R* is a crucial parameter for determining ionic conductivity (see the Supporting Material for details on calculation of the bulk electrolyte resistance (Equations (1) and (2))). Table 3 lists the fitted values of *R*. In addition to *R* and *Q*, the ionic conductivity of the GPE depends on the charge carrier concentration (*n*) and mobility (*μ*) [32,52].

*σ* = *nqμ* (4)

in which, *q* is the charge of mobile carriers.

According to the EIS results, the ionic conductivity was improved significantly upon incorporating 0.75 wt% FGNP and 1 wt% GNP, as compared to the Gel electrolyte sample. Further increase in the content of nanoplatelets, however, led to lower ionic conductivities. Furthermore, in all nanoplatelet-containing GPEs, the ionic conductivity of the FGNP-based GPEs exceeded that of the GNP-based one. The maximal ionic conductivity (4.11 mS cm^−1^) was recorded for the Gel–FGNP(0.75) sample. Such an enhancement could be justified by the increase in the count of charge carriers and ion transport pathways. As discussed earlier, owing to its hydrophilicity, FGNPs can be easily dispersed in polymers thanks to their numerous oxygen functional groups (PEG chains). In comparison with GNP, the FGNP possesses far broader interlayer spacing, which can be attributed to its surface functional groups (discussed in Section 3.1). Thus, the carbonyl groups of the PEG chains can adsorb the polar molecules. Besides, the FGNPs are arranged into a highly expanded and adjustable layered configuration, offering ion migration channels to produce homogenous pathways for ion carriers. Furthermore, the oxygen functional groups on the surface and edges can add to the physical interactions and facilitate not only the formation of the hydrogen bonds with polar groups of the PVDF–HFP, but also their intensive interaction with PEO due to their high compatibility with the PEG-grafted graphene. Proper dispersion of nanoplatelets through the matrix was also observed in the rheology tests, indicating the successful exfoliation of the FGNPs. The decrease in the crystallinity of the Gel–FGNP samples verified the physical crosslinking of the FGNPs with polymer chains encouraged the ion diffusion across the nanoplatelets’ surface and channels, giving rise to superior ionic conductivities [30,53]. The structure of the graphene-based gel electrolyte is schematically represented in Figure 6c to demonstrate the enhancement in the ion transport. Moreover, uniform dispersion of FGNPs could further promote the formation of a 3D network in the Gel, offering a continuous pathway for ionic conductivity, as elaborated in Section 3.3. The agglomeration of GNP-added samples occurred prior to that of the FGNP-containing ones. This could be explained by the presence of strong van der Waals and π–π interactions in the GNPs in contrast to the availability of more charge carrier ions in the FGNPs that facilitate the ion transport [45,54]. Based on the Nyquist data listed in Table 3, the maximum electrochemical performance was exhibited by Gel–FGNP(0.75) followed by Gel–FGNP(1), Gel–FGNP(0.5), and Gel–FGNP(1.5)). Above a given critical threshold [55], the aggregation of NPs reduced the charge transport efficiency by increasing the percolation threshold of redox species through the polymer chains. 

#### 3.5.2. Temperature-Dependent Ionic Conductivity of Gel Polymer Electrolytes

Temperature-dependent ionic conductivity assessments were performed at 30–100 °C to broaden our understanding about the ion transport mechanisms in the nanoparticle-containing GPEs. Arrhenius plots (Figure 6d) indicated the log (σ) of GPE samples against the inverse of temperature (1000/*T)*, indicating linear variation of the ionic conductivity. Therefore, the ionic conductivity can be defined by the Arrhenius equation, as follows [56]:

σ = σ_0_ exp(−E_a_/k_B_T) (5)

where σ_0_ is the pre-exponential frequency factor, *E*_a_ denotes the activation energy, *k*_B_ is Boltzmann’s constant (1.38 *×* 10^−23^ J K^−1^), and *T* represents the absolute temperature. This profile indicated the homogeneity of the Gel–FGNP sample (for the ion mobility phase) with no considerable polymer chain rearrangement with temperature. From another point of view, the diffusion and conductivity of charge carriers in the proposed GPE can be considered as hopping to the nearest energetically favorable empty (e.g., vacant, interstitial, and (sub)lattice) sites [57] throughout the host polymer matrix.

The mentioned model is not similar to the previously described conductive-mechanism models such as the Vogel–Tammann–Fulcher [58] model, in which the charge carrier diffusion is driven by the molecular movements of the polymer chains at higher temperatures, giving rise to the free volume within the host material. Even at elevated temperatures, the Arrhenius behavior of the presented GPEs was maintained, reflecting the structural contribution of the blend to restricting the segmental flexibility [55,59] of polymer chains in the matrix. Regarding the value of *E*_a_, Gel–FGNPs exhibited lower activation energies than Gel–GNPs, suggesting the role of the homogenous dispersion of FGNPs in the creation of the interstitial lattice sites between the polymer chains. These sites can further promote the ionic hopping in quasi-solid states. Therefore, the connection pathways provided by FGNPs were superior to those formed by the GNPs (as shown schematically in Figure 6c). The rise in *E*_a_ from 0.064 eV (Gel–FGNP(0.75)) to 0.065 eV (Gel–FGNP(1)) could be attributed to the enhanced percent weight of FGNPs, which could encourage the aggregation of larger nanocrystals and hence, hinder ion diffusion through disturbing the ions’ access to the vacancies in the host matrix (Table 4). The activation energy of the Gel sample was measured at 0.079 eV, reflecting the plasticization effect of the ILs, which led to a porous structure that facilitated the motion of the ions within the polymer film.

Conductivity of an electrolyte depends highly on diffusion-mediated ion movements and exchange reactions ($I^{-}/I_{3}^{-}$) between the redox species. In this case, the LiI/I_2_ electron exchange happens through a combination of electron hopping and bond exchange interactions, which can be described by a Grotthuss-type charge transfer. The fillers can facilitate the conduction mechanism. A similar Grotthuss charge exchange mechanism is involved in the conduction process by establishing a suitable transport channel for the redox couples. In the case of nanofiller-added polymer gel electrolytes, the existing cations (Li^+^) would be adsorbed onto the surface of the finely dispersed nanofillers; this would align the anionic redox couples through electrostatic interactions, giving rise to an electron transport pathway, and hence, facilitating the charge exchange process [55,60].

#### 3.5.3. Linear Sweep Voltammetry

Linear sweep voltammetry (LSV) tests (Figure 6e; Table 4) were conducted to examine the effective diffusion coefficient of triiodide ions (*D*_app_) to further verify the obtained EIS results. The sigmoid shape specifies the redox reaction happening at the electrode surface, i.e., $3I^{-}↔I_{3}^{-}+2e^{-}$. According to the primary mechanism underlying the dye-sensitized photovoltaics, $D_{app}^{I_{3}^{-}}$ is a major parameter for determining the diffusion coefficient of the triiodide ion, as follows:

(6) $$D_{app}^{I_{3}^{-}}=J_{lim}\frac{d}{2nFC}$$

in which, *J_lim_* shows the steady-state photocurrent density, *d* denotes the membrane thickness, and *n*, *F* and *C* are the number of electrons (i.e., *n* = 2), Faraday constant, and the concentration of $I_{3}^{-}$, respectively. 

Maximal *J*_lim_ was detected in Gel–FGNP(0.75) at a current density of 0.051 mA cm^−2^. This sample further exhibited the highest improvement in $D_{app}^{I_{3}^{-}}$, (from 2.48 × 10^−9^ cm^2^ s^−1^ for Gel to 8.14 × 10^−9^ cm^2^ s^−1^ for Gel–FGNP(0.75)). Such a considerable increase in the effective ion diffusion was a result of the highly amorphous matrix that was achieved upon adding FGNPs at the optimum dosage (0.75 wt%). Noteworthy, previous studies on GPE diffusion coefficient enhancement indicated mean diffusion coefficient values in the range of 10^−9^–10^−10^, highlighting the effectiveness of PEG functionalization for improving the GPE nanocomposite performance [61]. Further incorporation of FGNPs decreased the diffusion coefficient of GPE, which could be ascribed to the augmented crystallinity of the polymeric host as well as increased agglomeration of NPs. The ionic diffusion coefficients obtained from the steady-state voltammetry were consistent with the EIS data. The improved diffusion coefficient was caused by not only the polymer crystallinity and microstructural features of the GPE, but also the catalytic behavior of the GNPs. The incorporation of FGNP boosted the cation accumulation by the PEG chains, producing higher values of photocurrent and fill factor (as discussed in Section 3.6) [62]. 

### 3.6. Performance of Dye-Sensitized Solar Cells

In a DSSC, the dye molecules adsorbed on the surface of TiO_2_ are photoexcited before having the excited electrons transferred into the conduction band of the semi-conductive TiO_2_ photoanode. Afterward, the excited dye molecules are regenerated by redox couples (LiI/I_2_) before being reduced by the counter electrode [63]. Different parts of the device are schematically presented in Scheme S1.

#### 3.6.1. Efficiency of Dye-Sensitized Solar Cells 

**Figure 7:**
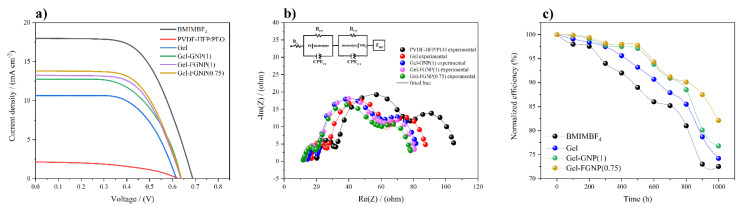
(**a**) *J–V* curves of the functionalized graphene DSSCs showing enhanced photovoltaic performance compared to the pristine graphene DSSCs, where the device containing Gel–FGNP (0.75) outperforms all other devices. (**b**) The Nyquist plots of PVDF–HFP/PEO, Gel, Gel–GNP(1), Gel–FGNP(1) and Gel–FGNP(0.75) electrolytes with the equivalent circuit in the inset. (**c**) Normalized conversion efficiencies of the DSSCs versus time.

*J–V* plots of the as-prepared DSSCs are presented in Figure 7a. Table 5 and Table S4 list the *J_SC_*, *V_oc_*, *FF*, and *η* values of the neat blend and nanocomposite GPEs, respectively. As can be seen, the *J_SC_* values were positively affected by the PEG-functionalization of the NP within the host matrix (to up to 13.81 mA cm^−2^ in the Gel–FGNP(0.75)). Besides, incorporation of 1 wt% FGNP influenced the *J_SC_* value negatively (13.24 mA cm^−2^). The device efficiency was, however, still higher than the one containing non-modified GNP(1) (12.74 mA cm^−2^). The increase in the *J_SC_* value could be attributed to the shortening of the ionic diffusion length resulting from the high catalytic activity of the GNPs in the chemical reduction of the ionic species throughout the system and further promotion of electron conductivity. However, the increase in the GNP/FGNP content beyond its percolation threshold would result in a 3D network across the NPs, which might impose some constraints on the pathways of the ions, thereby decreasing the photovoltaic performance of the device (see Figure S7). The efficiency of the PVDF–HFP/PEO electrolyte was calculated at 0.61%. Its photovoltaic performance, as well as other parameters, exhibited a drastic enhancement upon the incorporation of the IL, GNP, and FGNP, as compared to the blend electrolyte. The highest photoconversion efficiency (5.45%) was recorded with Gel–FGNP(0.75) in the presence of the masked FTO substrate at an open-circuit voltage of 0.637 V. Further incorporation of the FGNPs decreased the DSSC performance due to the resultant decrease in the ion transport and increase in the crystallinity.

As the designed DSSCs were fabricated and tested at the same temperatures with the same device components, the enhancement in *J_SC_* could be related to neither thermal acceleration of ions in the GPE nor the differences in photoanode properties. Indeed, the improvement in the dye regeneration kinetics, and hence, the *J_SC_* values was induced by the ion diffusion and charge transport efficiencies inside the electrolyte layer. Consequently, the increase in the *J_SC_* could be attributed to the ionic diffusion of the redox couple through the interconnected network. Such a phenomenon was a result of PEG functionalization through a Grotthuss-type electron hopping ion-exchange mechanism. It has been previously reported [37,59] that the exchange process ($I^{-}/I_{3}^{-}$) occurs in the GPE medium to facilitate the adsorption of redox-active species on the nanoparticle surface. The creation of concentrated and highly aligned zones promotes fast anion transport with almost no physical transfer of the liquid/solid. This mechanism operates in a similar way to the chemical bond exchange process that enables the proton transport within the aqueous media containing the Arrhenius acid [64]. The FGNPs seem to act in the same way, i.e., providing either the potential redox substrate or proper transport channel builder (via a Grotthuss-type charge transfer mechanism) to facilitate the dye regeneration by the ions. The chemical functionalization decreases the NP aggregation, retaining a relatively large active surface area on the nanoparticles. This would, in turn, align the local density of $I^{-}/I_{3}^{-}$ to further accelerate the diffusion of the redox couple across the interconnected network structure of the GPEs [62,65]. Enhancement in kinetics of the dye regeneration by increasing the mobilization of the charge carriers added to the values of *J_SC_*, *FF*, and *η*. 

#### 3.6.2. Internal Resistance of Dye-Sensitized Solar Cells

EIS analysis based on the so called Nyquist plots (Figure 7b) can further extend our insight by determining the interfacial processes affected by the FGNPs. The inset in Figure 7b shows the proposed device for this purpose in the form of an equivalent circuit. The experimental EIS data were fitted to the equivalent circuit followed by deriving the interface resistance values, as tabulated in Table S4 (see Supporting Materials for details (Equations (3)–(6))). In Figure 7b, the three individual arches, namely *R_PT_*, *R_CT_*, and *R_diff_* (refer to the real part of Z_diff_), are related to the charge transfer mechanism at the counter electrode/electrolyte and photoanode/electrolyte interfaces and the ion diffusion resistance for the redox couple ($I^{-}/I_{3}^{-}$) diffusion in the electrolyte, respectively. The x-axis intersection denotes the ohmic serial resistance (*R*_S_) of the FTO substrate. The EIS parameters support the conductivity nature of the PVDF–HFP/PEO blend and the Gel electrolyte samples in the presence/absence of the fillers. According to the EIS data, upon the incorporation of the fillers, both *R_PT_* and *R_CT_* followed initially decreasing trends followed by enhancements realized when the filler content reached an optimal value (Table S4). The reduction in *R_PT_* implies a fast reduction in the $I_{3}^{-}$ and creation of $I^{-}$ at the counter surface, while the decrease in the *R_CT_* is indicative of the ease of ion transport inside the electrolyte. The filler-containing ionic pathways facilitate the ion movement of $I^{-}$ and $I_{3}^{-}$ ions between the electrodes. As suggested by Figure 6c, in the presence of the NPs, the charge transport occurred with no obstacle through the specific pathway inside the electrolyte [60]. Filler incorporation increased the amorphous degree of the gel polymer electrolyte [66]. The addition of the filler into the GPEs increased the tendency of the electrolyte toward exhibiting a gel/liquid-like behavior, thereby lowering the resistance of the ions to the diffusion. The EIS results indicated lower *R_PT_* and *R_diff_* values for the Gel, Gel–GNP and Gel–FGNP samples in comparison with the PVDF–HFP/PEO. Nonetheless, filler incorporation did not cause any significant change in the *R_CT_* values of the DSSCs. As a measure of the diffusion resistance of the electrolyte, the value of *R_diff_* decreased in the filler-containing systems. The fast transport of the $I^{-}$ and $I_{3}^{-}$ was responsible for lower values of *R_diff_*. The lower values of *R_PT_* and *R_diff_* highlighted the improved performance of the filler-containing system compared to its filler-free counterpart.

#### 3.6.3. Stability of Dye-Sensitized Solar Cells

Last but not least, regarding the significance of the device stability and efficiency, the performance of the proposed functionalized graphene DSSC was assessed over time (Figure 7c). For this purpose, the performance of the best DSSC, i.e., “Gel–FGNP(0.75)”, was evaluated within a period of 1000 h and compared with those of homologous devices containing 1 wt% GNP, Gel, and pure IL (BMIMBF_4_). All devices were kept at room temperature under ambient conditions between the tests. The considerable performance retention of the FGNP-based DSSC was deduced to be related to the improved dispersion and better 3D network of the FGNPs, which decelerated the cell degradation and GPE collapse by means of the moisture-induced oxidized species (${IO}_{3}^{-}$). Therefore, besides the enhancement in overall device performance, the chemical functionalization contributed to the maintenance of the integrity and stability of the quasi-solid-state device.

The samples exhibited no degradation susceptibility during the tests, although a gradual degradation was evident as the time passed. For the Gel–FGNP(0.75) sample, the device efficiency was 82.19% after 1000 h. The decline in performance could be attributed to the creation of oxidized ions in the presence of moisture. Upon long-term exposure to the ambient atmospheric conditions, the $I^{-}/I_{3}^{-}$ couple is known to capture the atmospheric moisture and become oxidized [67], leading to a decrease in the ionic species content. The oxidized ionic species then fail to be reduced at the counter electrode. Upon long-term storage, the iodine could also sublimate, altering the ionic species content, and hence, disturbing the $I^{-}/I_{3}^{-}$ equilibrium.

## 4. Conclusions

PVDF–HFP/PEO-based polymer nanocomposites were investigated. According to the results, the ionic conductivity of the electrolyte and the DSSC performance were found to be highly dependent on the availability of free ions and incorporation of additives into the polymer electrolyte at optimal ratios. The mobility of the ions could be further affected by the free volume of the PVDF–HFP/PEO that could be enhanced by expanding the amorphous domains of the samples. As filler, FGNPs were incorporated into the PVDF–HFP/PEO and ionic liquid systems. The FGNP-grafted PEG molecules interacted with the oxygen atoms of the PEO chains through hydrogen bonding, thereby disrupting the blend crystallinity. DSC analysis indicated a decrease in the degree of crystallization upon the incorporation of the GNPs (and FGNPs, in particular). The strong polymer–nanoparticle interactions combined with proper connectivity improved the conductivity of the electrolyte significantly, lowering the required activation energy. The developed solar cells containing optimal dosage of FGNP (0.75 wt%) offered a *V_oc_* of 0.637 V and a *J_sc_* of 13.81 mA/cm^2^ and led to a solar energy conversion efficiency of 5.45% upon exposure to 100 mW/cm^2^. Based on the linear steady-state voltammetry results, the FGNPs could shorten the ionic diffusion length while enhancing the diffusion coefficient to about 8.14 × 10^−9^ cm^2^ s^−1^. The optimum DSSC exhibited superior stability as it succeeded to retain 82.19% of its original performance after 1000 h of storage, as per the results of regular periodic tests.

## Figures and Tables

**Table 1:** Sample codes along with their respective compositions.

Sample Name	Polymer Electrolyte	Constituents in Weight Percent
PVDF–HFP/PEO	Neat Blend	60/40
Gel	PVDF–HFP/PEO: IL: redox couple	42/28: 10: 20
Gel–GNP(x)^*^	“PVDF–HFP/PEO”: IL: redox couple: GNP	“42/28-x”: 10: 20: x
Gel–FGNP(x)	+PVDF–HFP/PEO”: IL: redox couple: FGNP	“42/28-x”: 10: 20: x

^*^ x refers to nanoparticle weight ratio (0.1, 0.25, 0.5, 0.75, 1, or 1.5 wt% with respect to blend).

**Table 2:** *T_g_*, *T_m_*, Δ*H* and degree of crystallinity (χ%) of the neat polymers, blend, and GPEs (A and B represent PVDF–HFP and PEO, respectively).

Sample	T_g_	Tm _A_	Tm _B_	ΔH_A_	ΔH_B_	χ% A	χ% Β	χ% An	χ% Βn
PVDF–HFP (A)	−21.54	141.51	-	28.74	-	27.45	-	-	-
PEO (B)	−41.41	-	67.23	-	126.52	-	59.20	-	-
PVDF–HFP/PEO	−24.77	140.4	66.58	14.38	35.21	22.90	41.19	-	-
Gel	−61.47	123.11	45.64	9.21	22.36	14.66	26.16	20.94	37.31
Gel–GNP(1)	−63.59	115.68	40.41	4.48	20.34	7.13	23.80	10.33	34.50
Gel–FGNP(0.75)	−66.07	110.48	36.73	3.71	19.41	5.90	22.71	8.52	32.79

n denotes the normalized amount by the blend weight ratio inside the sample.

**Table 3:** Ionic conductivities of Gel, Gel–GNP, and Gel–FGNP electrolyte membranes containing different amounts of nanoplatelets.

Sample Code	Graphene Type	Membrane Electrolyte Thickness (μ_m_)	R_b_ of the Electrolyte (ohm)	σ (mS cm^−1^)
Gel	-	283.71 ± 5	16.81 ± 0.24	2.15 ± 0.08
Gel–GNP(0.1)	Ordinary	344.27 ± 9	14.57 ± 0.41	3.01 ± 0.47
Gel–GNP(0.25)	Ordinary	337.88 ± 1	13.84 ± 0.85	3.11 ± 0.27
Gel–GNP(0.5)	Ordinary	325.57 ± 4	12.53 ± 0.86	3.31 ± 0.09
Gel–GNP(0.75)	Ordinary	286.00 ± 7	9.39 ± 0.04	3.88 ± 0.97
Gel–GNP(1)	Ordinary	216.74 ± 5	6.99 ± 0.63	3.95 ± 0.35
Gel–GNP(1.5)	Ordinary	221.39 ± 8	7.25 ± 0.95	3.89 ± 0.19
Gel–FGNP(0.1)	Functionalized	323.54 ± 3	13.21 ± 0.17	3.12 ± 0.41
Gel–FGNP(0.25)	Functionalized	304.91 ± 1	10.88 ± 0.76	3.57 ± 0.61
Gel–FGNP(0.5)	Functionalized	302.33 ± 4	9.85 ± 0.41	3.91 ± 0.58
Gel–FGNP(0.75)	Functionalized	271.34 ± 5	8.41 ± 0.63	4.11 ± 0.79
Gel–FGNP(1)	Functionalized	299.75 ± 2	9.57 ± 0.11	3.99 ± 0.31
Gel–FGNP(1.5)	Functionalized	309.71 ± 8	10.41 ± 0.58	3.79 ± 0.59

**Table 4:** Diffusion coefficient and activation energy of GPEs.

GPE	Graphene Type	$D_{app}^{}$/ 10^−9^ cm^2^ s^−1^	E_a_^*^/eV
Gel	-	2.48	0.079
Gel–GNP(1)	Ordinary	6.53	0.070
Gel–FGNP(1)	Functionalized	7.55	0.065
Gel–FGNP(0.75)	Functionalized	8.14	0.064

^*^ Activation energy determined from the Arrhenius plot (Equation (5)).

**Table 5:** Photovoltaic performance and equivalent circuit parameters fitted to EIS data of DSSCs from the Nyquist plot.

DSSC^*^	V_OC_ (V)	J_sc_ (mA cm^−2^)	FF (%)	ɳ (%)	R_s_ (Ω)	R_PT_ (Ω)	R_CT_ (Ω)	R_diff_ (Ω)
BMIMBF_4_	0.688 ± 0.002	17.98 ± 0.01	59.63 ± 0.003	7.38 ± 0.11	-	-	-	-
PVDF–HFP/PEO	0.622 ± 0.017	2.13 ± 0.04	47.31 ± 0.015	0.62 ± 0.09	20.16 ± 0.15	11.41 ± 0.38	45.11 ± 0.29	28.7 ± 0.36
Gel	0.617 ± 0.005	10.64 ± 0.04	61.51 ± 0.007	4.03 ± 0.06	16.9 ± 0.38	8.95 ± 0.32	38.3 ± 0.21	24.27 ± 0.31
Gel–GNP(1)	0.638 ± 0.010	12.74 ± 0.06	58.89 ± 0.014	4.78 ± 0.13	13.81 ± 0.25	6.35 ± 0.27	39.92 ± 0.42	23.14 ± 0.37
Gel–FGNP(1)	0.637 ± 0.002	13.24 ± 0.03	61.69 ± 0.011	5.20 ± 0.11	12.17 ± 0.28	7.5 ± 0.21	40.08 ± 0.25	21.01 ± 0.22
Gel–FGNP(0.75)	0.637 ± 0.005	13.81 ± 0.02	61.95 ± 0.006	5.45 ± 0.09	11.84 ± 0.17	6.81 ± 0.14	39.48 ± 0.11	20.2 ± 0.16

^*^ Average values over three measurements.

## Supplementary Materials

The following are available online at https://www.mdpi.com/2073-4360/12/7/1443/s1, Figure S1: FT-IR spectra of PEG, Figure S2: Raman spectra of GNP, GNP-COOH, and FGNP, Figure S3: Storage modulus versus strain amplitude, Figure S4: (a) complex viscosity, (b) Storage and loss modulus, (c) Storage modulus, (d) Loss modulus, and e) damping factor against angular frequency of neat PVDF-HFP, neat PEO, and PVDF-HFP/PEO blend at 190 °C, Figure S5: Differential pore size distributions obtained for polymer membranes, Figure S6: Schematics of the structures of (a) polymer blend, (b) Gel electrolyte, (c) Gel-GNP(1), and (d) Gel-FGNP(0.75), Figure S7: J–V curves of Gel-FGNP DSSCs containing (0.1, 0.25, 0.5, 0.75, 1, and 1.5 wt% FGNP), Table S1: EDX elemental composition of GNP, GNP-COOH, and FGNP nanoparticles, Table S2: Tg, Tm, ΔH and degree of crystallinity (χ%) of the neat polymers, blend, and GPEs (A and B, represent PVDF-HFP and PEO, respectively), Table S3: Comparison of the BET data of PVDF-HFP/PEO, Gel, Gel-GNP(1), and Gel-FGNP(0.75) membrane films, Table S4: Photovoltaic Performance and Equivalent-Circuit Parameters Fitted to EIS Data of DSSCs from Nyquist plot, Scheme S1: Schematic of the DSSC.
