# Supplementary material for: Microstructural Development and Rheological Study of a Nanocomposite Gel Polymer Electrolyte Based on Functionalized Graphene for Dye-Sensitized Solar Cells
Source: Polymers (Basel). 2020 Jun 27;12(7):1443. doi: 10.3390/polym12071443 (PMC7408189; doi:10.3390/polym12071443)
Supplement: Supplementary file 1 [file polymers-12-01443-s001.pdf]

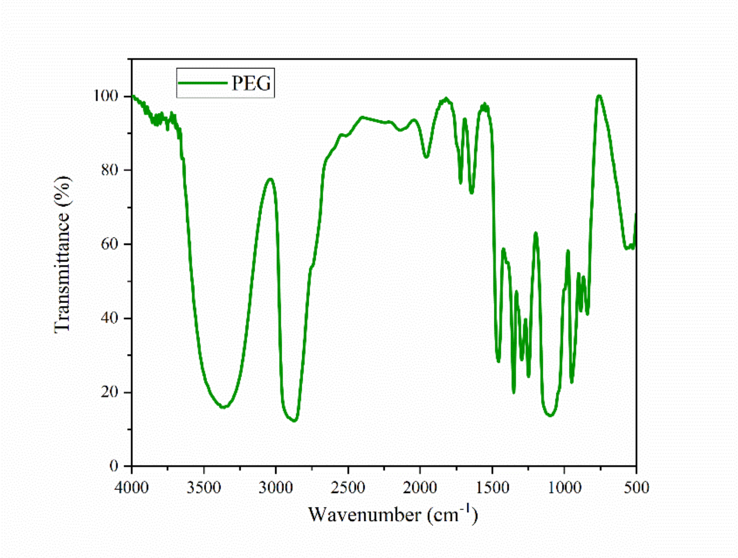

Figure S 1 FT-IR spectra of PEG.

FT-IR spectra of PEG is shown in Figure S *1*.

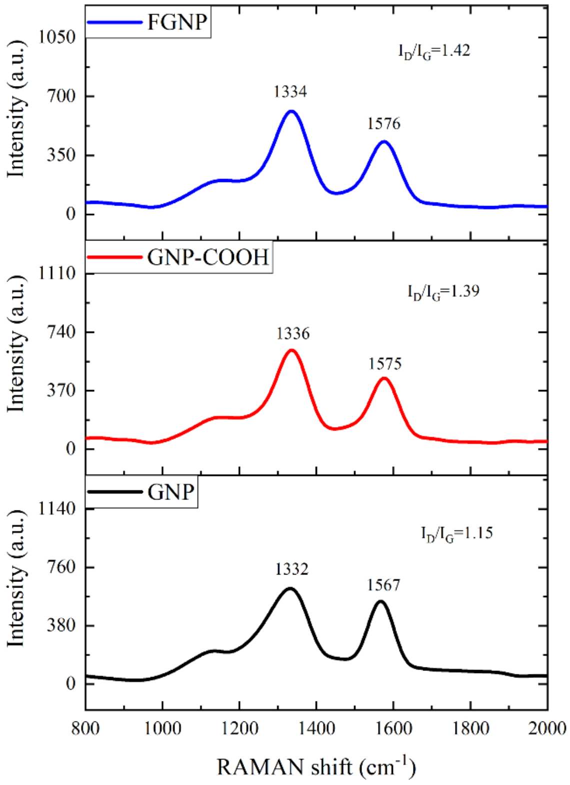

Figure S 2 Raman spectra of GNP, GNP-COOH, and FGNP.

Raman analysis has been reported as a robust tool for the characterization of the GNP functionalization reaction. Raman spectrum of GNP, GNP-COOH, and FGNP systems are presented in

Figure S 2. Two characteristic peaks of carbon allotropes (D and G bands) can be detected. The D-band emerged at ~1340-1360 cm^{-1} reflecting the ring-breathing mode of sp^2 atoms. The D-band is specifically an indicator of the irregularities or the defect. The emergence of G band at 1567 cm^{-1} indicated the bond stretching in all pairs of sp^2 atoms (either rings or chains); it manifested itself as the prominent mode in GNP [1]. It is well known that the ratio of D band intensity to that of G band (I_D/I_G) is under the influence of irregularity patterns of graphene nanoplatelets. Acid treatment of GNP and chemical grafting of PEG to COOH-GNP are two main reactions that lead to the formation of some defects on the surface of GNP and disturb the NPs' pattern. In the case of our samples, I_D/I_G ratio of GNP, COOH-GNP, and FGNP was determined 1.15, 1.39, and 1.42, respectively. An increment in I_D/I_G ratio of FGNP is a sign of PEG chemical grafting onto GNP [2].

An important technique for the quantitative determination of the GNP functionalization reaction is energy-dispersive X-ray analysis (EDX). The main elements, as well as their concentrations, are depicted in Table S 1 for GNP, GNP-COOH, and FGNP. According to the results, only carbon and oxygen atoms were present as the main elements of all the samples and no impurity was found. A comparison between the GNP and GNP-COOH nanoparticles revealed an increase in the oxygen content (from 2.1 to 10.1 wt%) after treatment with acids. Higher oxygen contents were also detected in FGNP (14.2 wt%) due to the presence of grafted PEG chains possessing one single oxygen atom for each repeating unit of the polymer. The mentioned characterization techniques were conducted to confirm the occurrence of the chemical reaction and efficient progress of the PEG grafting.

Table S 1 EDX elemental composition of GNP, GNP-COOH, and FGNP nanoparticles.

Sample	Carbon (wt%)	Oxygen (wt%)	O/C ratio
GNP	97.9	2.1	0.021
GNP -COOH	89.4	10.1	0.113
FGNP	85.7	14.2	0.165

1. **CHNO elemental analysis**

The results of elemental analysis as another powerful technique evidenced that functionalization reaction performed very well. The oxygen content before and after the acid treatment was 3.39% and 11.25%, respectively. Increments of oxygen content in the samples after acid treatment can be an indication of surface oxidation of graphene nanoplatelets and existence of oxygen-containing groups. Higher oxygen contents were also identified in FGNP (15.9 wt%) due to the existence of grafted PEG chains owning one single oxygen atom for each repeating unit of the polymer.

Table S 2 Tg, Tm, ΔH and degree of crystallinity (χ%) of the neat polymers, blend, and GPEs (A and B, represent PVDF-HFP and PEO, respectively).

**Sample**	**T_g**	**Tm $_A$**	**Tm $_B$**	**ΔH$_A$**	**ΔH$_B$**	**χ% A**	**χ% B**
Gel-GNP(0.1)	-61.52	121.73	45.33	9.01	22.21	14.34	25.98
Gel-GNP(0.25)	-61.87	120.59	44.26	8.13	22.01	12.94	25.75
Gel-GNP(0.5)	-62.68	118.17	43.18	6.26	21.57	9.96	25.23
Gel-GNP(0.75)	-63.43	116.44	41.83	5.08	20.76	8.09	24.29
Gel-GNP(1)	-63.59	115.68	40.41	4.48	20.34	7.13	23.80
Gel-GNP(1.5)	-63.36	117.02	42.11	5.89	20.98	9.38	24.54
Gel-FGNP(0.1)	-61.54	121.55	44.85	8.74	22.09	13.91	25.84
Gel-FGNP(0.25)	-62.62	118.46	41.57	6.96	21.18	11.08	24.78
Gel-FGNP(0.5)	-65.89	114.09	38.31	4.98	20.85	7.93	24.39
Gel-FGNP(0.75)	-66.07	110.48	36.73	3.71	19.41	5.90	22.71
Gel-FGNP(1)	-65.98	113.81	38.81	3.99	20.96	6.35	24.52
Gel-FGNP(1.5)	-63.81	115.53	41.29	5.06	21.37	8.05	25.00

2. **Determination of the linear viscoelastic region**

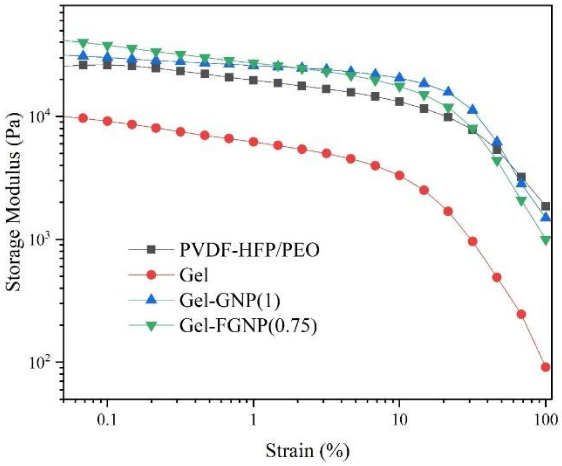

Figure S 3 Storage modulus versus strain amplitude.

For differentiating the linear viscoelastic region from the nonlinear, investigating the dispersive stability of NPs and the influence of IL, the strain amplitude sweep tests were carried out under the controlled frequency (1 rad s^{-1}) and the strain range of 0.05–100%. The storage modulus was plotted as a function of the strain amplitude for PVDF-HFP/PEO blend, Gel and its nanocomposites including GNP and FGNP as shown in Figure S *3*. The linear-nonlinear transition of the viscoelastic behavior occurred at lower strain values in the Gel, Gel-GNP(1), and Gel-FGNP(0.75) as compared with the neat blend.

As can be seen, modulus reduction happens because of plasticizing effect of IL in the Gel sample. It is observed that modulus reduction in Gel sample happens as a result of a) plasticizing effect of IL, and b) decreasing of physical crosslinking density. It should be noted that reduction of modulus, which happens by these two phenomena are in competition with modulus improvement causes by gelation interactions. Thus, the linearity of the viscoelastic behavior was reduced and the storage modulus of the Gel sample was rapidly decreased after the critical strain. Also, this transition in the FGNP containing nanocomposites occurred at lower strains in comparison with pristine nanoplatelets. Such an observation could be assigned to the microstructural variations due to the presence of the functionalized graphene nanoplatelets and the breaking of several essential elastic linkages in the 3D framework of the nanoplatelets whose contents are more dominant in the functionalized nanoplatelets. Additionally, storage modulus exhibited a rapid decline in the Gel-FGNP(0.75) implying more robust 3D network structures when FGNPs are used instead of GNPs. Therefore, more linear rheological assessments were conducted within the linear viscoelastic region (at 0.5% amplitude).

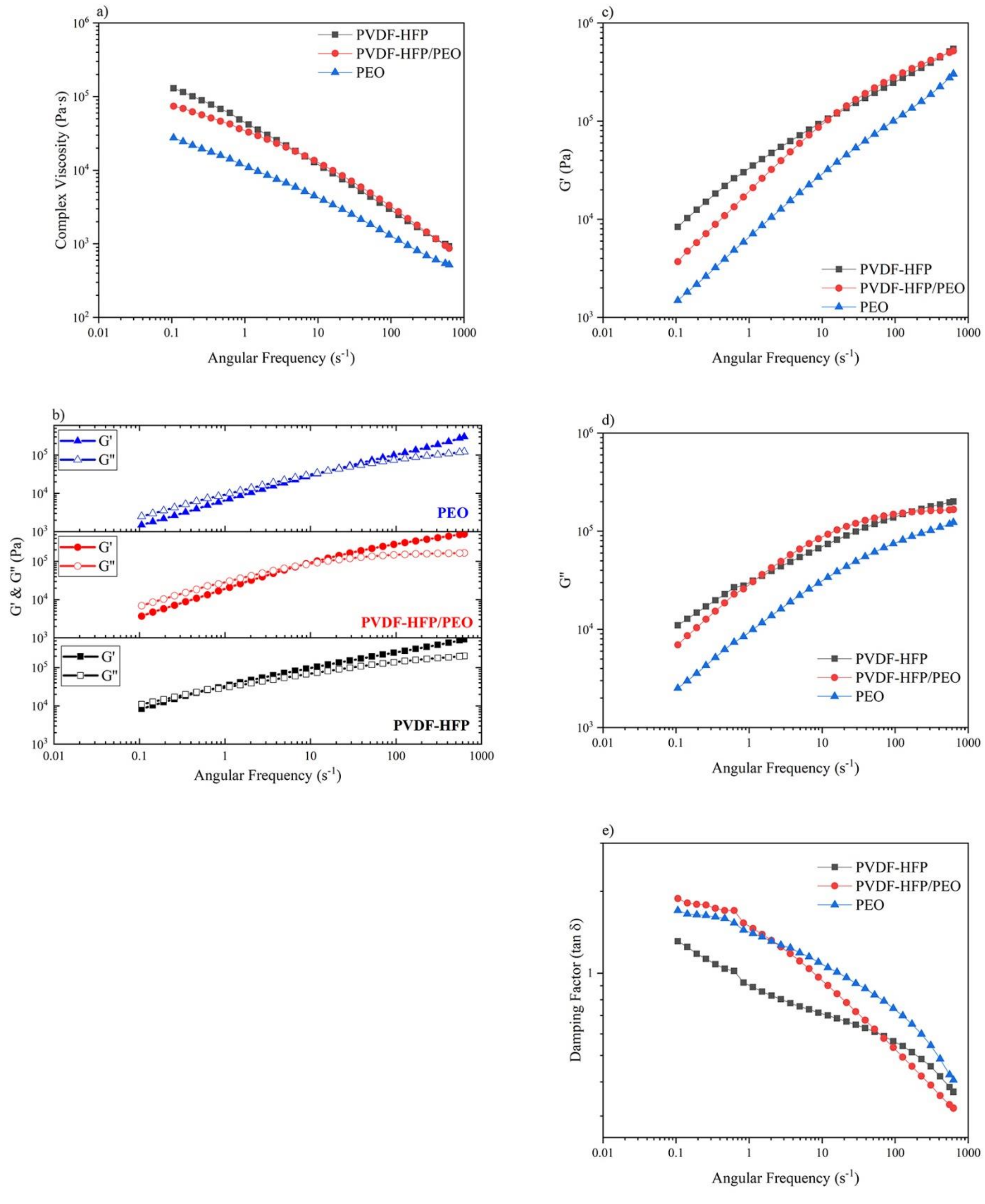

Figure S 4 a) complex viscosity, b) Storage and loss modulus, c) Storage modulus, d) Loss modulus, and e) damping factor against angular frequency of neat PVDF-HFP, neat PEO, and PVDF-HFP/PEO blend at 190 °C.

Figure S 5 shows the pore size distributions of the PVDF-HFP/PEO, Gel, Gel-GNP(1), and Gel-FGNP(0.75) membrane films, using the mercury intrusion porosimetry analysis. Based on the results, the PVDF-HFP/PEO membrane exhibited a slight increase in average pore size with addition of IL in the polymer blend. The pore size of PVDF-HFP/PEO varied in the range of 0.2- 2.5 μm, with most of the pores ranging within 0.6- 1.5 μm in size, and in Gel sample most of the pores ranging within 0.6- 2.2 μm in size. However, effect of graphenes in the matrix is more pronounced. It can be observed that presence of whether GNP or FGNP in the polymer matrix lead to rapid increase in pore size within 4- 10 μm.

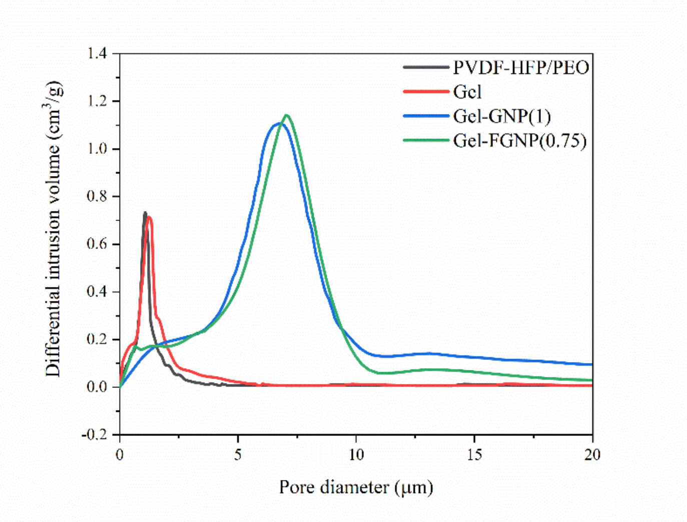

Figure S 5 Differential pore size distributions obtained for polymer membranes.

The BET measurements was conducted to study the pore radius of the membrane films (

Table S 3). It seems pore size of the membranes affected by addition of graphene. Pore size of membranes containing nanoparticles is larger than that of PVDF-HFP/PEO, Gel samples. However, the effect of functionalization of GNP on the pore radius is insignificant. Membrane films of PVDF-HFP/PEO and Gel samples possess smaller pores (0.81 and 0.86 μm, respectively) than Gel-GNP(1) and Gel-FGNP(0.75) (15.25 and 15.32 μm, respectively).

Table S 3 Comparison of the BET data of PVDF-HFP/PEO, Gel, Gel-GNP(1), and Gel-FGNP(0.75) membrane films.

**GPE**	**Pore radius (μm)**
PVDF-HFP/PEO	0.81
Gel	0.86
Gel-GNP(1)	15.25
Gel-FGNP(0.75)	15.32

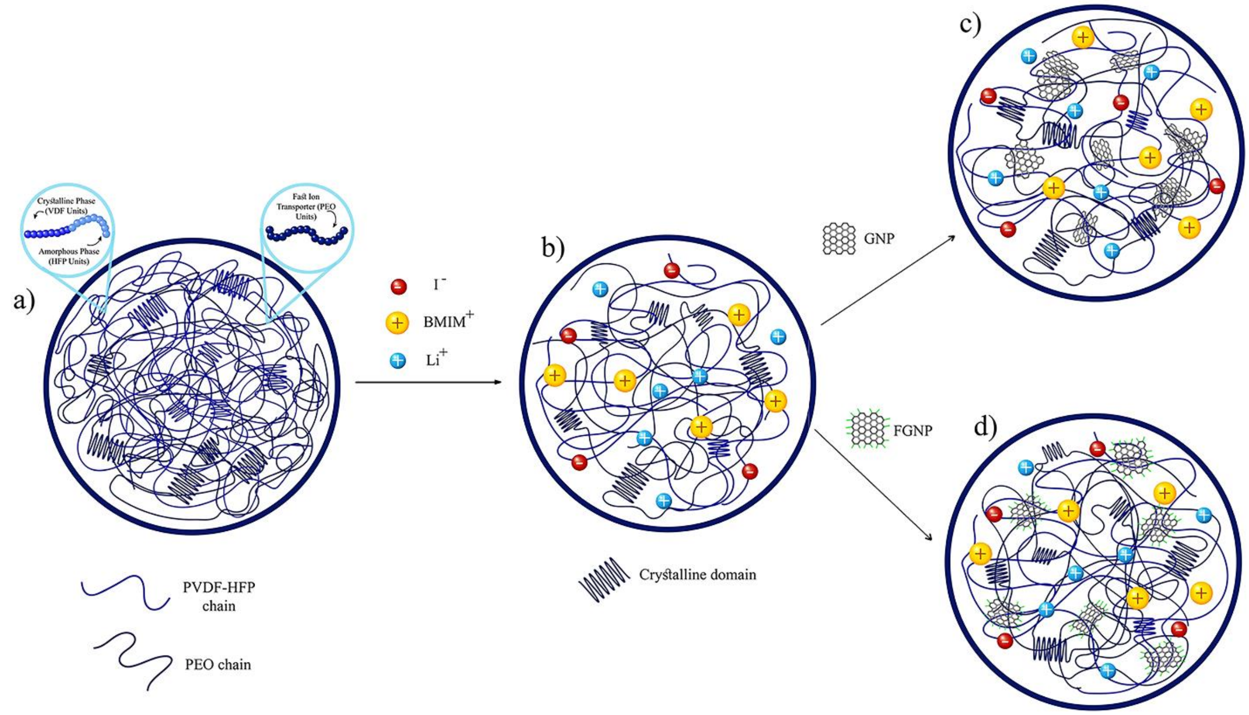

Figure S 6 Schematics of the structures of (a) polymer blend, (b) Gel electrolyte, (c) Gel-GNP(1), and (d) Gel-FGNP(0.75).

Figure S 6 schematically represents the structural diagrams of four different polymeric membranes. Figure S 6-a presents the intermolecular and intramolecular reactions of the polymer blend chains as the result of the microcrystalline regions and dipole-dipole interaction between the blend chain constituents. These interactions are the consequence of the electrostatic interaction occurring between the fluorine atom of a PVDF-HFP chain and the hydrogen atom of the other PVDF-HFP chain (intermolecular) as well as the interactions between CF_2 groups of PVDF–HFP and C–O–C species of PEO (intramolecular). PVDF-HFP has a semicrystalline character; thus, its mechanical properties and thermal stability are high enough. However, regarding the insulating feature of most polymers including PVDF-HFP, they are practically useful. The addition of PEO (as an ion-conductive polymer) can reduce the crystallinity and improve the ion conductivity of the blend by increasing the amorphous domain of the matrix. Also, the incorporation of ionic liquid as plasticizer and redox couple (LiI/I_2) offers more available ions for transportation making it a proper candidate for electrochemical purposes. In addition, presence of pores in the polymer film further promoting ion movements and thus, ion conduction [3]. At the same time, better $BMIMBF_4$ and BF_4 as an IL complex [3] interactions can decrease the entanglement density of the polymer chains resulting in a porous configuration due to plasticizing effect of IL as illustrated by Figure S 6-b. The schematic diagrams of Gel-GNP(1), and Gel-FGNP(0.75) samples are given in Figure S 6(c and d), respectively. These figures demonstrate the effect of nanoplatelets in increasing the porosity by forming larger pores. The polymer surrounded connecting network of the graphene improved the electrical features, conductivity, and thermal stability.

3. Calculation of the bulk electrolyte resistance of GPEs

The impedance behavior was explored by connecting, in series, the bulk electrolyte resistance (R_b) to a constant phase element (Q) along a circuit. The real and imaginary parts of the impedance are given as follows [4]:

$$Z' = R_b + \frac{cos\,(\frac{\pi p}{2})}{k^{-1}\omega^p} \quad (1)$$

$$Z'' = \frac{sin\,(\frac{\pi p}{2})}{k^{-1}\omega^p} \quad (2)$$

where Z′ and Z″ are the real and imaginary parts of the impedance, respectively, R_b represents the bulk resistance for the membrane electrolyte, ω is the angular frequency ($\omega = 2\pi f$ where f is the frequency in Hz), k^{-1} denotes the capacitance of the Q, and p is the angle formed between the spike and the horizontal axis on the Cole-Cole plot. In the case of GPEs, the bulk resistance (R_b) would be much important for estimating the ionic conductivity as the intercept of the Nyquist plot (Z′ vs. Z″) along the real axis mainly at higher frequency region.

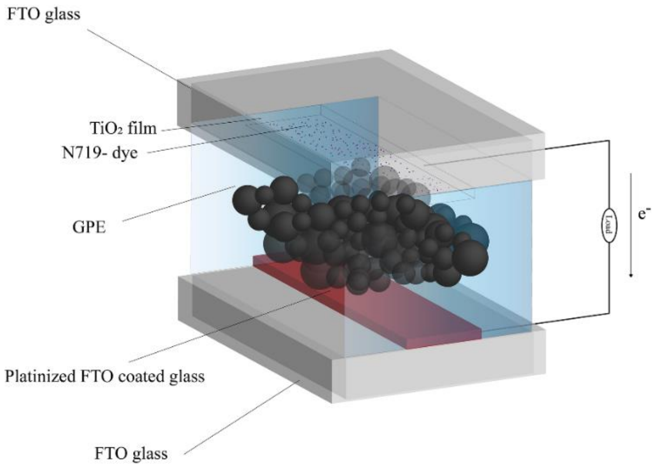

Scheme S 1 Schematic of the DSSC.

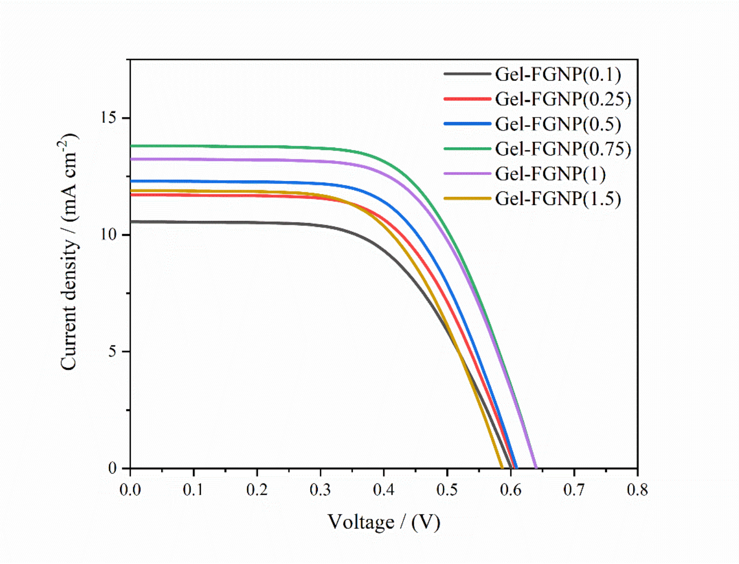

Figure S 7 J–V curves of Gel-FGNP DSSCs containing (0.1, 0.25, 0.5, 0.75, 1, and 1.5 wt% FGNP).

Table S 4 Photovoltaic Performance and Equivalent-Circuit Parameters Fitted to EIS Data of DSSCs from Nyquist plot.

DSSC*	V_{OC} (V)	J_{sc} (mA cm^{-2})	FF (%)	η (%)	R_s (Ω)	R_{CT1} (Ω)	R_{CT2} (Ω)	R_{CT3} (Ω)
**Gel-FGNP(0.1)**	0.601±0.015	10.58±0.04	58.70±0.014	3.73±0.07	16.14±0.16	8.05±0.29	38.21±0.25	23.04±0.38
**Gel-FGNP(0.25)**	0.610±0.017	11.79±0.06	59.59±0.017	4.29±0.05	14.51±0.51	7.82±0.31	38.14±0.11	22.41±0.24
**Gel-FGNP(0.5)**	0.614±0.011	12.34±0.06	60.97±0.016	4.62±0.13	12.73±0.44	7.03±0.38	39.86±0.38	21.38±0.35
**Gel-FGNP(0.75)**	0.637±0.005	13.81±0.02	61.95±0.006	5.45±0.09	11.84±0.17	6.81±0.14	39.48±0.11	20.2±0.16
**Gel-FGNP(1)**	0.637±0.002	13.24±0.03	61.69±0.011	5.20±0.11	12.17±0.28	7.5±0.21	40.08±0.25	21.01±0.22
**Gel-FGNP(1.5)**	0.587±0.013	11.93±0.05	59.25±0.012	4.15±0.11	12.51±0.25	7.88±0.29	40.24±0.41	22.07±0.09

***Data is measured and averaged by three samples.**

4. **Resistance calculation**

As shown in Figure 7-b (the inset), one may combine the impedance elements Z_s, Z_{PT} , Z_{CT}, and Z_{diff} to develop an electrical equivalent circuit for a DSSC device [5–7]. Accordingly, the sum of (3) through (6) would give the DSSC impedance:

$Z = Z_s + Z_{PT} + Z_{CT} + Z_{diff}$

Such a circuit is then composed of the series resistance (R_s) and the photoanode, the electrolyte, and the counter electrode impedances (Z_{CT}, Z_{diff}, and Z_{PT}, respectively).

In a DCCS, the ohmic R_s is most significantly controlled by electrode substrate sheet resistance as well as the electrolyte resistance.

$$Z_s = R_s \quad (3)$$

Following another approach, CPE can be devised to describe the charge transfer phenomenon at the counter electrode and the respective impedance, Z_{PT}:

$$Z_{PT} = \frac{R_{PT}}{1 + (j\omega)^{n_{PT}} R_{PT} CPE_{PT}} \quad (4)$$

in which n_{PT} is the constant phase element (CPE) indices at the counter electrode/electrolyte,

Withholding information on the semiconductor layer characteristics in term of the electron transfer phenomenon and the recombination behavior of the semiconductor – electrolyte interface, the photoanode impedance controls the diffusion-reaction model [8]. The following expressions give the photoanode impedance [9]:

$$Z_{CT} = \frac{R_{CT}}{1 + (j\omega)^{n_{CT}} R_{CT} CPE_{CT}} \quad (5)$$

where n_{CT} corresponds to the photoanode/electrolyte interfaces.

$$Z_{diff} = R_{diff} \frac{tanh\left[(\frac{j\omega}{\omega_d^p})^{1/2}\right]}{(\frac{j\omega}{\omega_d^p})^{1/2}} \quad (6)$$

In the above equation, ω_d^p and R_{diff} represent the characteristic diffusion frequency and diffusion resistance of the ionic phase of the electrolyte, respectively.

References

1. Ferrari, A.C. Raman spectroscopy of graphene and graphite: disorder, electron–phonon coupling, doping and nonadiabatic effects. *Solid State Commun.* **2007**, *143*, 47–57.

2. Manafi, P.; Ghasemi, I.; Manafi, M.R.; Ehsaninamin, P.; Hassanpour Asl, F. Non-isothermal crystallization kinetics assessment of poly(lactic acid)/graphene nanocomposites. *Iran. Polym. J. (English Ed.* **2017**, *26*, 377–389, doi:10.1007/s13726-017-0527-z.

3. Vyas, M.K.; Chandra, A. Ion–Electron-Conducting Polymer Composites: Promising Electromagnetic Interference Shielding Material. *ACS Appl. Mater. Interfaces* **2016**, *8*, 18450–18461, doi:10.1021/acsami.6b05313.

4. Arof, A.K.; Naeem, M.; Hameed, F.; Jayasundara, W.J.M.J.S.R.; Careem, M.A.; Teo, L.P.; Buraidah, M.H. Quasi solid state dye-sensitized solar cells based on polyvinyl alcohol (PVA) electrolytes containing $$\mathbf{I}^{\mathbf{-}}/\mathbf{I}_{\mathbf{3}}^{\mathbf{-}}$$I-/I3-redox couple. *Opt. Quantum Electron.* **2014**, *46*, 143–154, doi:10.1007/s11082-013-9723-z.

5. Wang, Q.; Moser, J.-E.; Grätzel, M. Electrochemical Impedance Spectroscopic Analysis of Dye-Sensitized Solar Cells. *J. Phys. Chem. B* **2005**, *109*, 14945–14953, doi:10.1021/jp052768h.

6. Fabregat-Santiago, F.; Bisquert, J.; Palomares, E.; Otero, L.; Kuang, D.; Zakeeruddin, S.M.; Grätzel, M. Correlation between Photovoltaic Performance and Impedance Spectroscopy of Dye-Sensitized Solar Cells Based on Ionic Liquids. *J. Phys. Chem. C* **2007**, *111*, 6550–6560, doi:10.1021/jp066178a.

7. Selvanathan, V.; Yahya, R.; Alharbi, H.F.; Alharthi, N.H.; Alharthi, Y.S.; Ruslan, M.H.; Amin, N.; Akhtaruzzaman, M. Organosoluble starch derivative as quasi-solid electrolytes in DSSC: Unravelling the synergy between electrolyte rheology and photovoltaic properties. *Sol. Energy* **2020**, *197*, 144–153, doi:https://doi.org/10.1016/j.solener.2019.12.074.

8. Bisquert, J. Theory of the Impedance of Electron Diffusion and Recombination in a Thin Layer. *J. Phys. Chem. B* **2002**, *106*, 325–333, doi:10.1021/jp011941g.

9. Bisquert, J.; Garcia-Belmonte, G.; Fabregat-Santiago, F.; Ferriols, N.S.; Bogdanoff, P.; Pereira, E.C. Doubling exponent models for the analysis of porous film electrodes by impedance. Relaxation of TiO2 nanoporous in aqueous solution. *J. Phys. Chem. B* **2000**, *104*, 2287–2298.
